# Spatial distribution of tuberculosis from 2002 to 2012 in a midsize city in Brazil

**DOI:** 10.1186/s12889-016-3575-y

**Published:** 2016-09-01

**Authors:** Mirna de Abreu e Silva, Cláudia Di Lorenzo Oliveira, Rafael Gonçalves Teixeira Neto, Paulo Augusto Camargos

**Affiliations:** 1 Municipal de Saúde, Av. Getúlio Vargas 268, 35500-024 Divinópolis, MG Brazil; 2 Campus Centro Oeste Dona Lindu, Universidade Federal de São João Del-Rei, Rua Sebastião Gonçalves Coelho 400, Chanadour, 35501-296 Divinópolis, MG Brazil

**Keywords:** Tuberculosis, Epidemiology, Spatial-temporal analysis, Spatial distribution, Kernel density map

## Abstract

**Background:**

Tuberculosis (TB) remains a major public health problem in many developing countries. Exploratory spatial analysis is a powerful instrument in spatial health research by virtue of its capacity to map disease distribution and associated risk factors at the population level. The aim of the present study was to describe the epidemiologic characteristics and spatial distribution of new cases of TB reported during the period 2002–2012 in Divinopolis, a midsized city located in the state of Minas Gerais, southeastern Brazil.

**Methods:**

Sociodemographic and clinical data relating to the study cases were retrieved from the national Brazilian database and geocoded according to residential address. Choropleth and kernel density maps were constructed and a spatial-temporal analysis was performed. Tracts defined by the 2010 national census were classified as sectors with higher or lower densities of new TB cases based on the kernel density map. Multivariate logistic analysis was used to compare the two types of sectors according to income, level of literacy and population density.

**Results:**

A total of 326 new cases of TB were reported during the study period. Residential addresses relating to 309 (94.8 %) of these were available in the SINAN database and the locations were geocoded and mapped. The average incidence of TB during the study period was 14.5/100,000 inhabitants. Pulmonary TB was the most predominant form (73.6 %) and 74.5 % of patients had been cured. The percentage of cases was highest in males (67.8 %) and individuals aged 25–44 years (41.1 %), and lowest in children aged less than 15 years (4.6 %). The disease was spatially distributed throughout the urban district. The incidence rate among urban census tracts ranged from 0.06 to 1.1 %, and the disease occurred predominantly in the downtown area (99.3 %). Higher population density was associated significantly with increased odds of living in a sector with a “higher density of cases”, even after adjusting for income and education (odds ratio = 13.7).

**Conclusions:**

The highest density of cases was strongly associated with higher population density but not with lower income or level of literacy.

## Background

Tuberculosis (TB) remains major public health issue especially in low and middle income countries. According to the World Health Organization (WHO), there were an estimated 9 million new cases of TB in 2013 with 1.5 million deaths and some 3 million cases not diagnosed or not reported [1]. The disease is more prevalent among males than females and in adults than in children, with the majority of cases occurring in individuals aged between 15 and 59 years [1].

The worldwide prevalence of TB is related to social inequality, poverty, overcrowding, migration and the inefficiency of TB control programs [2]. In this regard, 82 % of the 2013 estimated incident cases were concentrated in 22 countries, among which Brazil was ranked 16th with respect to the total number of cases and 22nd considering incidence rate, prevalence and mortality [1].

In Brazil, all cases of TB must be reported to the SINAN for inclusion in the governmental database [3]. The SINAN system, which commenced operation in 1997, holds details of all cases of selected diseases according to the list published by Brazilian Ministry of Health. Case reports are transmitted to SINAN via standardized forms that include home address, clinical and laboratory data and information concerning treatment applied. Tabulated data, without patient identification or home address, are available on the Internet, although full access to the database is restricted to authorized personnel.

Divinópolis is a midsized Brazilian city located in the center-west of the state of Minas Gerais at a distance of 104 km from Belo Horizonte, the state capital. The current TB control program in Divinópolis is based on active case finding of patients exhibiting respiratory symptoms that may be associated with the disease. Once identified, patients are requested to supply two sputum samples (one at the time of suspicion of TB and the second on the following day) for acid-fast bacillus (BAAR) test and to undergo a chest X-ray. If the BAAR test is positive, culture is requested to identify the *Mycobacterium* but treatment for TB is started immediately and is free for all patients [3]. Currently, 21 patients are receiving treatment for TB in Divinópolis (13 diagnosed in 2015 and 6 in 2016) but, of these, only one patient is undergoing supervised short-course directly observed therapy (DOTS).

Mapping TB cases allows the detection of spatial clusters, and such information can be useful in understanding socio-economic differences among neighborhoods, in facilitating preventative measures and in developing more efficient and focused policies for the elimination of the disease [4–6]. Computer-based Geographical Information System (GIS) tools have become especially useful in identifying clusters of areas with the highest incidence of a disease, in assessing the evolution of the spatial distribution of disease with respect to time and in analyzing health care networks [7, 8]. In the present study, we have analyzed the spatial distribution of TB cases in Divinopolis, and investigated the association between density of cases, as defined by spatial analysis, and sociodemographic variables.

## Methods

### Study setting

The descriptive and spatial study was conducted in the municipality of Divinópolis, Minas Gerais, Brazil, encompassing a land area of 708.115 km^2^. According to the 2010 census conducted by the IBGE [9], the population of Divinópolis was 213,016 and the mean population density was 300.82 inhabitants/km^2^ distributed over 295 census tracts, which it is defined the minimal areas of analysis for our study.

### Data collection

The study included all new cases of pulmonary and extra pulmonary TB in Divinópolis that had been reported to SINAN during the period 2002–2012. Data concerning gender, age, clinical form and clinical evolution of the disease for each patient were retrieved from the SINAN database. The UTM coordinates of the patient’s residence were determined from the home address recorded in the database using Google Earth™ software version 7.15 (Alphabet Inc., Mountain View, CA, USA). A Choropleth map was constructed, with the aid of ARCGIS^®^ version 10.1 software (Esri, Redlands, CA, USA), by superimposing map points generated for TB cases geocoded at the home level on to a digital map of the metropolitan area marked with polygons representing the IBGE 2010 census tracts [9].

### Data analysis

In order to identify the presence of spatial-temporal clusters, the observed and expected incidence rates were compared for each of the census tracts. The spatial-temporal analysis was performed with the aid of Statscan^®^ software (Statscan Inc., Wisconsin, USA) using the space-time permutation scan statistic proposed by Kulldorff et al. [10].

A smooth kernel density map of TB cases map was employed in the visual identification of areas exhibiting the highest numbers of cases/m^2^ of surface. This statistical smoothing technique allowed filtering for the variability of the data set while retaining the essential characteristics of the data locations [11]. A search radius equal to 125.5 m was employed in the preparation of the kernel density map. Census tracts were then classified into two mutually exclusive categories according to the density of TB cases. If the tract was mostly (about 60 %) in one of the higher density areas it was classified as a sector with a “higher density of cases”, otherwise it was deemed to be a sector with a “lower density of cases”. Multivariate logistic regression analysis was performed in which the dependent variable was the kernel map density classification where sectors with a “higher density of cases” were assigned a value of 1 and all other sectors were assigned a value of zero. Variables were subsequently selected for the multivariate model according to the crude odds ratio (*p* <0.20). The independent variables included in the model were population density, percentage literacy and percentage of residents receiving one Brazilian minimum wage or less. Population density was grouped according to quartiles and digitized as follows: 0 for sectors with <2.12 inhabitants/m^2^, 1 for sectors with 2.12–5.27 inhabitants/m^2^, 2 for sectors with 27–8.3 inhabitants/m^2^ and 3 for sectors with > 8.3 inhabitants/m^2^. Regression analysis was carried out with the aid of STATA^®^ software version 13.0 (StataCorp, College Station, TX, US).

## Results

**Fig. 1:**
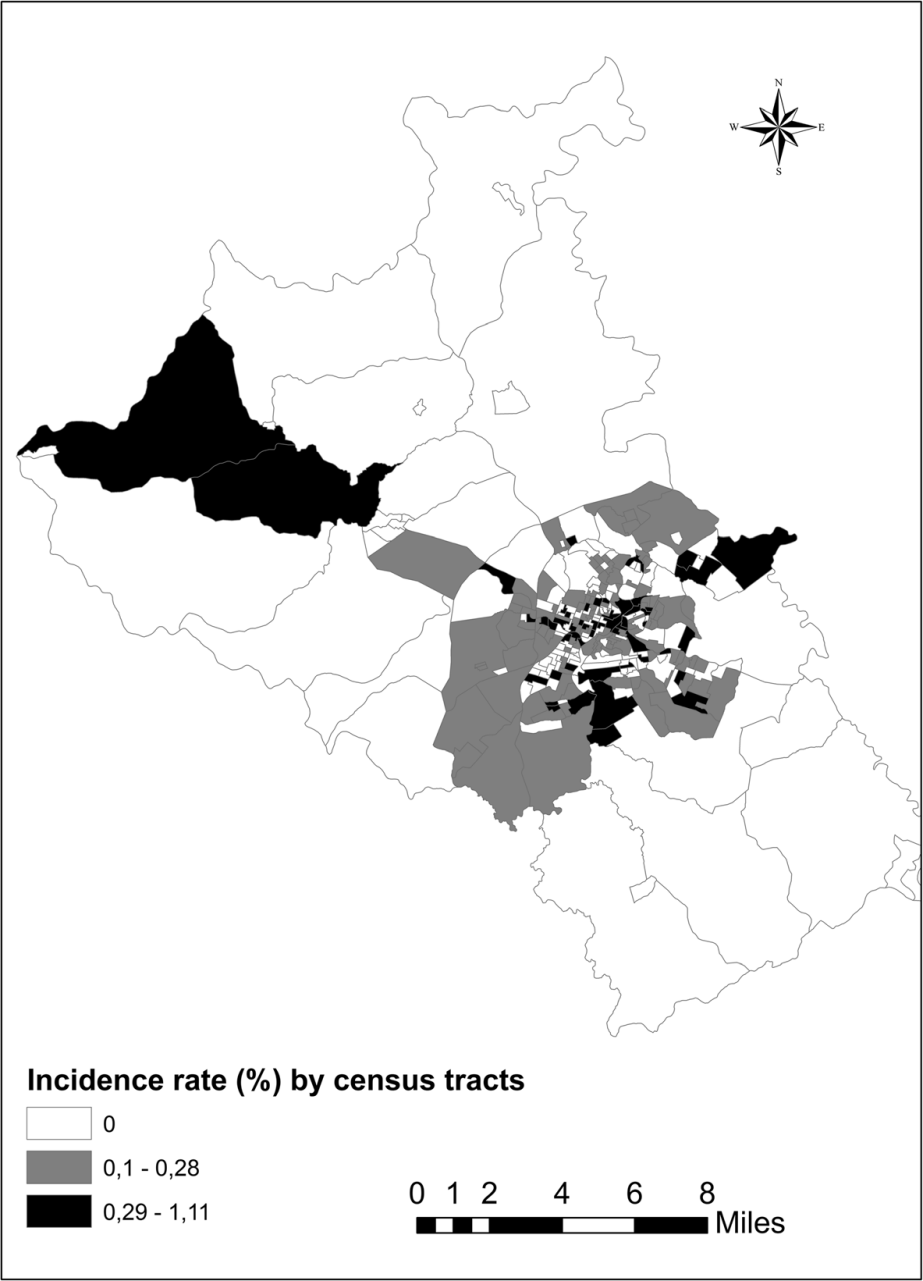
Incidence rate (%) of tuberculosis in Divinópolis, MG, Brazil, from 2002 to 2012distributed according to census tract

A total of 387 cases of pulmonary and extrapulmonary TB in Divinópolis had been reported to SINAN during the period 2002–2012. Among these reports, 326 (84.2 %) represented new cases, the sociodemographic and clinical characteristics of which are shown in Table 1, with an average incidence of 14.5/100,000 inhabitants. Three-hundred-and-nine (94.8 %) new cases were associated with complete residential addresses in the SINAN database, and these were geocoded and subsequently mapped (Fig. 1).

**Table 1:** Sociodemographic and clinical characteristics of new cases of tuberculosis in Divinópolis during the period 2002–2012

Variable	Number	Percent
Gender		
Male	221	67.8
Female	105	32.2
Age group (years)		
0–14	15	4.6
15–24	35	10.7
25–44	134	41.1
45–64	110	33.8
≥ 65	32	9.8
Clinical form of tuberculosis		
Pulmonary	240	73.6
Extrapulmonary	77	23.6
Both	9	2.8
Clinical evolution		
Cure	243	74.5
Dropout	40	12.3
Death from other causes	30	9.2
Death from tuberculosis	8	2.5
Transfer	5	1.5

Data extracted from the SINAN database [3]

Cases of TB had been reported in 163 (55.2 %) of the 295 census tracts constituting the municipality, with one case of the disease reported in 77 (47.2 %) tracts, two cases in 46 (28.2 %) tracts, three cases in 28 (17.2 %) tracts, four cases in 6 (3.7 %) tracts, five cases in 4 (2.5 %) tracts and six cases in 2 (1.2 %) tracts. The disease had occurred predominantly in urban tracts (99.3 %), most especially in the downtown area, with crude incidence rates per tract ranging from 0.06 to 1.1 %. The very few cases that had been reported in rural tracts were scattered over relatively large areas, the maps of which were lacking in relevant detail. For these reasons, only urban census tracts were included in the spatial analysis.

**Fig. 2:**
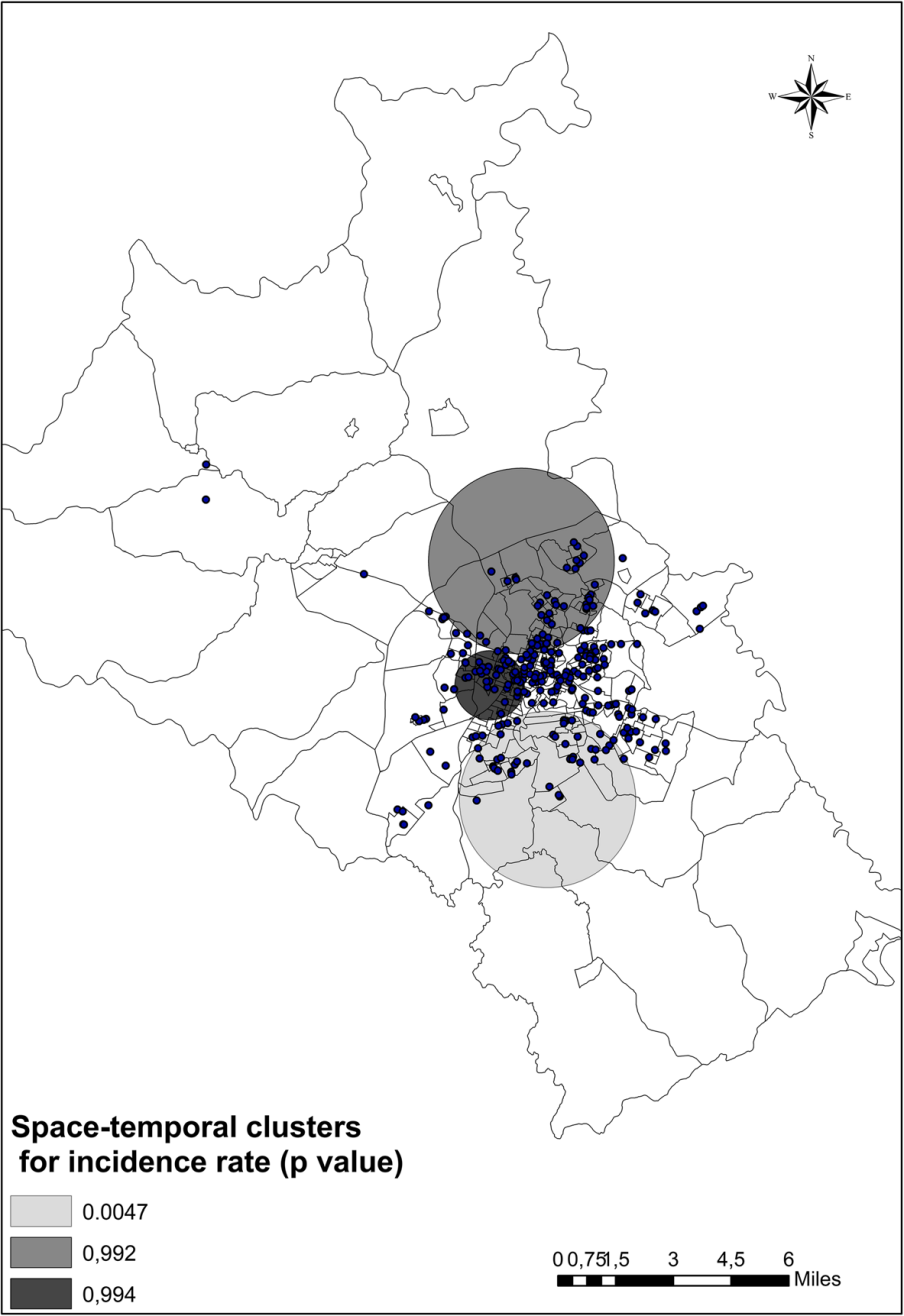
Spatial-temporal clusters of new cases of tuberculosis in the urban area of Divinópolis, MG, Brazil from 2002–2012. The *p*-values indicate the significance of the reduction in incidence rate according to time-window for each cluster

The space-time analysis indicated the presence of three major clusters (Fig. 2), one of which exhibited a statistically significant difference (*p* <0.0047). This cluster, which was located in the southern region of the city, presented a cluster in space and in the time window from 2003 to 2004. During this period, a significantly smaller number of cases were observed in the cluster region in comparison with other geographical areas.

**Fig. 3:**
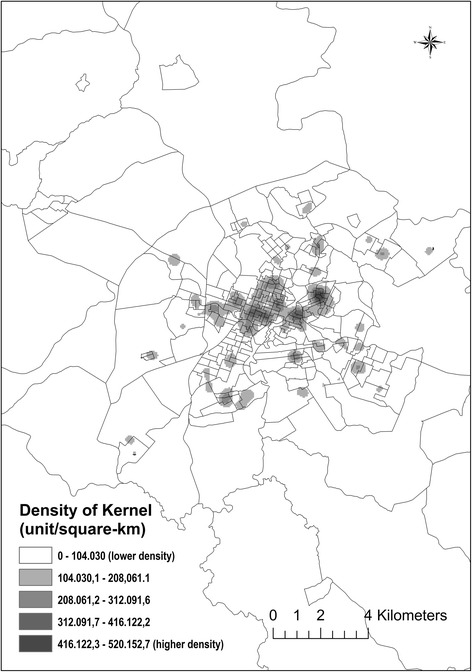
Kernel density map showing the distribution of new cases of tuberculosis in the urban area of Divinópolis, MG, Brazil from 2002–2012

The kernel density map for the distribution of new cases is shown in Fig. 3 in which areas with higher numbers of TB cases are represented by darker shading. The spatial distribution of the disease within the city was heterogeneous during the study period, with the highest densities being found in the central area of the city (downtown).

**Fig. 4:**
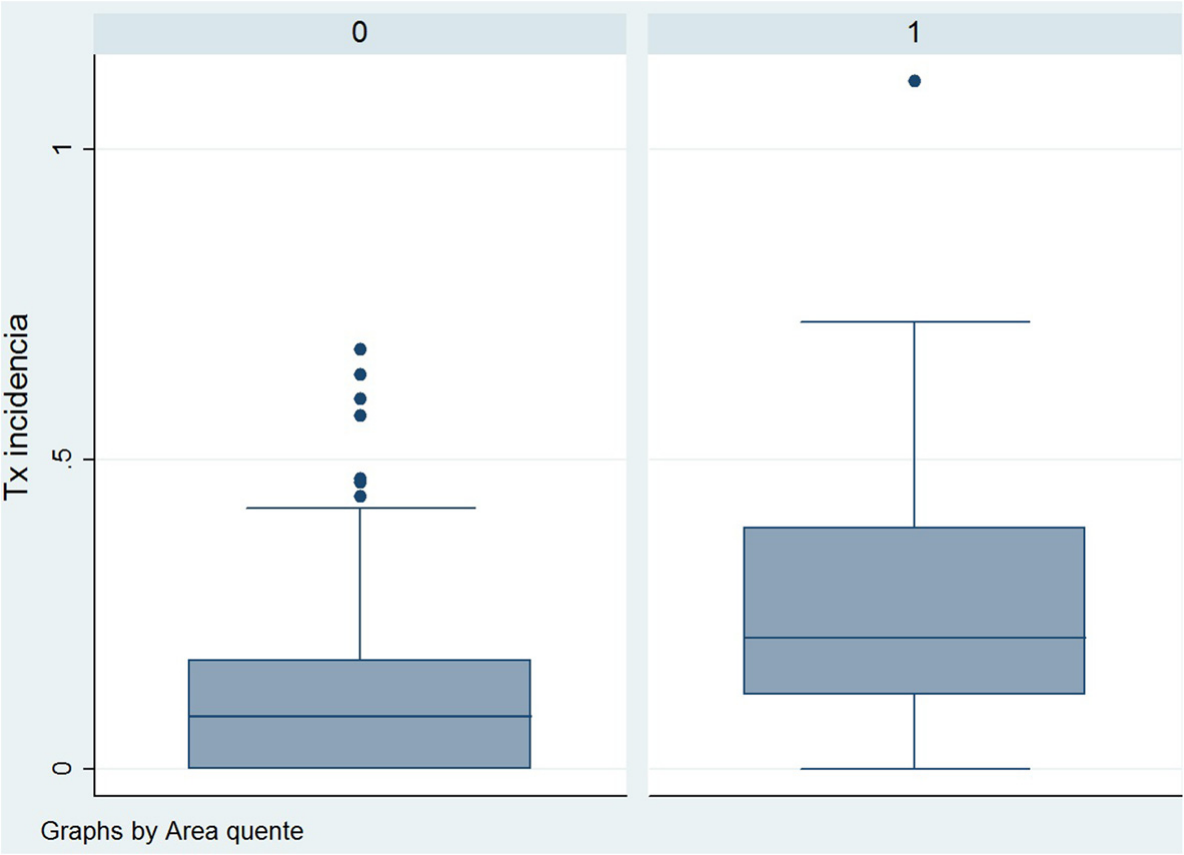
Box plots of incidence rates in sectors of Divinópolis, MG, Brazil, classified as having lower or higher densities of cases of tuberculosis during the period 2002–2012

Box plots (Fig. 4) of incidence rates revealed that data collected from sectors with a “higher density of cases” (*n* = 71) exhibited amore heterogeneous distribution about the median and significantly higher median and interquartile range values in comparison with data relating to sectors with a “lower density of cases” (*n* = 205).

Crude and adjusted odds ratios were calculated in order to compare sectors presenting a “highest density of cases” with those presenting a “lower density of cases”. Neither income nor percentage literacy was associated with “higher density of cases”. Higher population density was associated significantly with increased odds of living in a sector with a “higher density of cases” (Table 2), although incidence rate and population density were not correlated (Pearson correlation coefficient = -0.027).

**Table 2:** Crude and adjusted odds ratios comparing sectors with higher and lower densities of TB cases

Variable	Crude odds ratio (95 % confidence interval)	Adjusted odds ratio (95 % confidence interval)
Income (≤ one minimum salary)	0.97 (0.95–0.99)	0.98 (0.95–1.08)
Percentage literacy	1.03 (0.94–1.1)	0.93 (0.81–1.05)
Population density^a^		
0	1.0	1.0
1	2.98 (0.9–9.8)	2.7 (0.82–9.28)
2	8.0 (2.6–24.7)	7.5 (2.4–23.5)
3	14.7 (4.8–44.7)	13.7 (4.4–42.8)

^a^Population density ranges: 0 < 2.12 inhabitants/m^2^; 1 = 2.12–5.27 inhabitants/m^2^; 2 = 5.27–8.3 inhabitants/m^2^; 3 > 8.3 inhabitants/m^2^

## Discussion

In Divinópolis, the pulmonary manifestation of TB predominated over the extrapulmonary form. The average incidence of TB in the municipality during the study period fulfilled the Millennium Development Goal of <25.6 cases/100,000 inhabitants by 2015 as recommended by WHO [1]. Although the observed incidence rate was lower than the mean value for the whole of Brazil [12], this does not necessarily indicate success of the preventative measures applied. First, the number of patients identified with respiratory symptoms that could be associated with TB was relatively low, meaning that suspected cases of the disease were fewer than would be expected. Secondly, a previous study conducted by our group in the same municipality established that health workers did not follow the protocol for diagnosis for TB properly [13]. Therefore, possible explanations for the lower incidence rate of TB in the area may be under-diagnosis or under-reporting of the disease.

The observed predominance of TB among males in the economically active age group (25–44 years) may be related to the lifestyle characteristics of this segment of society such as increased prevalence of human immunodeficiency virus (HIV) and alcoholism, among others, that favor the disease [14, 15].

A number of spatial studies have associated higher incidence rates of TB with higher housing density, poorer living conditions and lower socioeconomic factors [6, 16–18]. In the present study we evaluated the density of cases from the kernel density map and hypothesized that density, income and percentage literacy of the population could partly explain the differences observed.

The disease was spatially distributed throughout the urban district without significant predominance in any specific area. The census tracts in the downtown areas with higher numbers of cases were those where the population was particularly overcrowded. Our results indicated that population density was the factor most associated with a higher density of TB cases. This finding was as expected since larger numbers of inhabitants imply increased risk, but the risk was strongly associated with population density (inhabitants/m^2^) and was independent of income and percentage literacy. Conversely, income and literacy were not associated with a higher density of cases or with a higher incidence rate.

According to Clark et al. [6], higher housing density is associated with a greater risk of contracting TB, while overcrowding increases the odds of being infected significantly. Contemporary cities are characterized by complex interactions comprising social networks and community connections that extend globally. These interactions carry important implications for health, particularly with respect to emerging and reemerging diseases, and reinforce the idea that inhabitants are constantly affected by city dynamics [19]. Moreover, population density and lack of urban planning can exacerbate existing social vulnerabilities such as the lack of sanitation, inadequate housing, overcrowded public transport and overloaded health services. Our results would suggest that population density plays an important role in the spatial determination of TB even after adjustment for income.

The present study was subject to a number of limitations. Firstly, while it is obligatory to notify SINAN about every case of TB, it is highly likely that some cases may not have been reported. Secondly, residential addresses were either missing or incomplete for around 5 % of the reported cases and these could not be geocoded. Such cases most probably occurred in areas with the lowest socioeconomic conditions. In addition, each of census tracts was considered homogeneous with respect to household density and it was not possible to include intra-household density even though this would be a variable of considerable interest.

## Conclusion

The mapping of cases is a convenient tool for the spatial characterization of TB, the results of which promote a better understanding of the distribution of the disease and the areas in which the risk of infection is highest. In the period of study, the sectors of Divinópolis presenting the highest density of cases were located in the central region of the city. The most important factor associated with high case density was high density of the population. It is concluded, therefore, that the odds of exposure to TB infection might be the result of a number of issues associated not only with the individual but also with population dynamics and/or socioeconomic conditions. Spatial and temporal distribution data provide the input required to elaborate an explanatory chain relating to the problems of the area and facilitate specific intersectoral action, thereby contributing to decision-making for the improvement of TB control activities.

## Abbreviations

BAAR: Acid-fast bacillus
DOTS: Short course directly observed therapy
GIS: Geographic information system
HIV: Human immunodeficiency virus
TB: Tuberculosis
SINAN: Notifiable diseases information system
IBGE: Brazilian Institute of Geography and Statistics
